# What is the yield of EBUS-TBNA for re-evaluation of previously treated non-small-cell lung cancer?

**DOI:** 10.55730/1300-0144.5619

**Published:** 2023-02-13

**Authors:** Ayperi ÖZTÜRK, Tuğba ÇİÇEK, Aydın YILMAZ

**Affiliations:** 1 Department of Interventional Pulmonology, Atatürk Sanatoryum Training and Research Hospital, Health Science University, Ankara, Turkey; 2 Department of Chest Diseases, Konya Numune Hospital, Konya, Turkey

**Keywords:** Endobronchial ultrasound, nonsmall cell lung cancer, reevaluation

## Abstract

**Background/aAim:**

Locoregional recurrence in lung cancer still remains an important problem. We aimed to indicate the effectiveness of endobronchial ultrasound-guided transbronchial needle aspiration (EBUS-TBNA) for reevaluation in previously treated nonsmall cell lung cancer (NSCLC).

**Materials and methods:**

NSCLC patients who underwent EBUS for rebiopsy of suspicious recurrent or progressive lesions between January 2010 and June 2017 were reviewed. Patients were categorized into two groups based on the previous treatment modalities: Group 1 (G1) consisted of patients who had been treated with chemoradiotherapy, and Group 2 (G2) consisted of patients who had undergone radical surgery.

**Results:**

Of 115 patients, 100 patients enrolled in the study. Of 26 patients with 35 lymph nodes in G1, malignant cells were identified in thirteen patients (50%). Anthracosis was detected in the remaining. Malignancy was detected in 28 patients (37.8%) in G2. Thirty-three patients were diagnosed as benign (24 anthracosis; 8 lymphocytes, and 1 granulomatous); 8 were not sampled, and inadequate material was obtained in five. The sensitivity, specificity, negative and positive predictive value, and overall diagnostic accuracy of EBUS-TBNA for rebiopsy in G1 were 84.8%, 100%, 89.1%, 100%, and 93.2%, respectively. These values were all perfect in G2.

**Conclusion:**

EBUS-TBNA could be preferred as a feasible and efficient procedure for rebiopsy in previously treated NSCLC patients.

## 1. Introduction

Lung cancer remains the most frequently diagnosed and lethal type of cancer worldwide [1]. Although surgery for early-stage nonsmall cell lung cancer (NSCLC) is performed as a curative treatment, tumor recurrence is the most common cause of treatment failure after surgery. Overall survival rates for localized and locoregional NSCLC have been reported at 92% and 46%, respectively. In addition, five-year survival rates ranged from 73% to 91% after surgery in pathologic stage 1 NSCLC, 58% to 66% in stage II NSCLC, and 47% in stage IIIA NSCLC [2,3]. Moreover, recurrence rates after a complete curative surgical resection ranged from 30% to 75% [4]. As a result, the American College of Chest Physicians has recommended that patients who undergo curative-intent surgical resection for NSCLC should also undergo chest computed tomography (CT) every 6 months for the first 2 years after resection and every year thereafter [5]. According to pathology results, surgery might be combined with adjuvant chemotherapy and/or radiotherapy, which has the potential to damage the lung parenchyma and mediastinum (e.g., inflammation or fibrosis). On thorax CT; inflammation, fibrosis, and infectious complications are indicated by mediastinal nodal enlargement and lung parenchymal abnormalities. Therefore, a differential diagnosis should be performed to determine whether recurrence has occurred.

Positron emission tomography (PET) with CT has been used as a tool to determine recurrence; however, high false-positive rates, in addition to low specificity, negative predictive values (NPV), and low diagnostic accuracy, have been associated with this method [6,7]. In addition, surgical techniques, such as mediastinoscopy (MS) and mediastinotomy, have also been used. However, the sensitivity and specificity of MS are lower in restaging cancer. Furthermore, some difficulties limit the use of MS, such as surgical trauma and difficulty in reexamination because of adhesions and fibrosis, which are induced by the initial procedure and induction treatment [7, 9]. Moreover, MS is unable to access the hilar lymph nodes and inner lungs around the bronchus. Endobronchial ultrasound-guided transbronchial needle aspiration (EBUS-TBNA) is a minimally invasive technique that is currently preferred as the first procedure in the mediastinal nodal staging of lung cancer [10,11]. EBUS-TBNA has a similar yield in comparison with MS for the mediastinal staging of NSCLC [12], and some reports have suggested that EBUS-TBNA is superior [13]. In recent years, there has been an increase in the number of studies that have examined the diagnostic accuracy of EBUS for restaging the mediastinum after neoadjuvant therapy in stage IIIA-N2 NSCLC patients. However, the amount of research on postsurgical local recurrence remains insufficient [14–21]. Therefore, in this study, we examine the ability of EBUS-TBNA to diagnose locoregional recurrence in both surgically treated NSCLC patients and medically inoperable patients who have received chemoradiotherapy.

## 2. Material and Methods

### 2.1. Patient selection criteria

This single-center, retrospective study with prospective follow-up was approved by the Local Ethics Committee of the Atatürk Chest Diseases and Thoracic Surgery Training and Research Hospital, (2656/23) and all the participants signed the written consent. All EBUS-TBNA procedures were applied between January 2010 and June 2017.

The inclusion criteria for this study were as follows: a) NSCLC patients who had been surgically treated, b) NSCLC patients who were surgical candidates but who were medically inoperable and who had received chemotherapy and/or chemoradiotherapy, c) patients with negative results for lymph node metastasis by EBUSTBNA verified by invasive techniques (MS, surgery, etc.), and patients with at least 1-year follow-up by thorax CT and /or PET-CT.

Also, the exclusion criteria were formed as: a) with stage IV NSCLC patients, b) those who applied after neoadjuvant therapy, c) those who had non-NSCLC pathological results, and d) those who had no follow-up in our hospital.

### 2.2. Procedures

The EBUS-TBNA test was applied in an operating room by two experienced interventional pulmonologists using a convex EBUS probe (BF-UC180F; Olympus, Tokyo, Japan) with a dedicated scanner (EU-ME1; Olympus, Tokyo, Japan) on patients under deep sedation by midazolam and propofol, commonly practiced transorally. After all stations were checked by EBUS, the lymph nodes were sampled according to their malignant sonographic features (i.e. roundness, heterogeneity, presence of coagulative necrosis, distinctive limits, and hypoechoicity), regardless of size. In addition, all lymph nodes with SUV-max values >2.5 mm and >10 mm in PET-CT were also sampled; 22-gauge needles (NA-201SX-4022, Olympus) were used. Between three and six aspiration passes were made at each lymph node in all patients. No major complications were observed, except in two patients who had a fever after the procedure.

### 2.3. Cytological examination

Cell blocks and cytological specimens were prepared for every sampled lymph node. The cytological materials were first air-dried and then stained with May–Grunwald–Giemsa and hematoxylin and eosin before examination. Cell blocks were prepared by flushing the material in 10 cc of saline solution. They were then immediately transferred to the pathology department. After the specimens were placed in 10% formalin, the samples were embedded in paraffin, and 6-micron sections of each sample were obtained. Immunohistochemical stains were performed to classify the tumors. Thyroid transcription factor-1 (TTF-1), napsin A, and p40 were used to discriminate primary lung cancer. Rapid on-site cytopathological evaluation (ROSE) was not performed.

If the sample contained lymphocytes and granulomas, it was grouped as benign, while those containing neoplastic cells were grouped as malignant. In addition, samples lacking lymphocytes were defined as inadequate materials in the study.

### 2.4. Statistical analysis

Since all EBUS-TBNA cases applied for restaging in a particular period were included in this study retrospectively, a power analysis was not performed beforehand. Statistical analyses were conducted using Predictive Analytics Software (PASW) Statistics for Windows (SPSS Inc. Version 18.0, Released 2009; Chicago, IL, USA). The descriptive data were defined as the mean standard deviation or median (minimum–maximum) values for the continuous variables and as percentage values for the categorical variables. The sensitivity, specificity, diagnostic accuracy, and positive and negative predictive values (PPVs, NPVs) were calculated for EBUS-TBNA as follows: sensitivity (True positive (TP)/ [TP + False negative (FN)]), specificity (True negative (TN)/ [TN + False positive (FP)]), PPV (TP/[TP + FP]), NPV (TN/[TN + FN]), and diagnostic accuracy ([TP + TN]/total patients).

## 3. Results

One hundred and fifteen patients satisfied the inclusion criteria and underwent EBUS-TBNA for the restaging of NSCLC. Fifteen patients were excluded, including patients who had no follow-up (n = 9), patients who applied after neoadjuvant therapy (n = 3), and patients who had other pathological results (n = 3). Therefore, 100 patients participated in the study. The patients were divided into two groups: Group 1 (26%) included the medically inoperable group and patients who had undergone EBUS-TBNA after chemotherapy and/or chemoradiotherapy; Group 2 (74%) included patients who had undergone EBUS-TBNA after curative surgery. In Group 1, the mean age was 63.9 (±9.9); in Group 2, the mean age was 62 (±7.9). The individual characteristics of the patients in Group 1 and Group 2 are shown in Table 1.

Thirty-five lymph nodes from 26 patients in Group 1 and 103 lymph nodes from 74 patients in Group 2 were sampled. All samples from the patients in Group 1 contained lymphocytes; however, in Group 2, five patients (6.8%) lacked inadequate levels of lymphocytes. These five patients declined the advanced invasive procedure and were followed up clinically and radiologically. In both groups, the most frequently sampled lymph nodes were in the right lower paratracheal station, which was sampled in 46.2% and 35.1% of Groups 1 and 2, respectively, and in the subcarinal station, which was sampled in 46.2% and 32.4% of Groups 1 and 2, respectively. The median number of sampled lymph nodes was 1 (range, 1–3) in both groups, except for eight patients in Group 2, who showed no pathologic lymph nodes. In addition, the number of aspiration passes ranged from three to six for all patients. No serious complications were related to EBUS-TBNA. The characteristics of the lymph nodes in Group 1 and Group 2 are displayed in Table 2. In Group 1, among 26 patients with 35 lymph nodes, malignant cells were identified in 13 patients (50%); however, anthracosis was detected in the remaining patients. In Group 1, the median radiological and clinical follow-up time was 13.2 ± 9.1 months (Table 1). In Group 2, no recurrence was observed during follow-up. Malignancy was detected in 28 patients (37.8%). Moreover, 33 patients were diagnosed as benign. This group included a positive diagnosis of anthracosis in 24 patients (32.4%), reactive lymph nodes (lymphocytes) in eight patients (10.8%), and granulomatous diseases in one patient (1.4%) at 20.5 (±15.4) month follow-up. Eight patients were not sampled because they had millimetric and irregular lymph nodes, and inadequate material was obtained from five patients, and no recurrence was observed during follow-up. The outcomes for all patients in Group 1 and Group 2 are summarized in Figure.

The results of EBUS-TBNA for restaging in NSCLC are shown in Table 3. The sensitivity, specificity, NPVs, PPVs, and overall diagnostic accuracy of EBUS-TBNA in detecting locoregional recurrence in patients with previously surgically treated lung cancer were 84.8%, 100%, 89.1%, 100%, and 93.2%, respectively. These values were all satisfactory in the medically inoperable group. No complications were observed during the study.

## 4. Discussion

Locoregional recurrence in lung cancer after both curative surgery and chemoradiotherapy remains an important problem [4]. Because of difficulties due to adhesions and fibrosis in reevaluating mediastinum by MS, high false-positive rates, low specificity, NPV, and diagnostic accuracy of PET/CT due to postinflammatory mediastinal changes after surgery, fibrosis, and infectious complications, the diagnosis of recurrence is very difficult [7–8,22]. A few previous studies have reported the diagnostic performance of EBUS-TBNA for restaging NSCLC [14–21, 23]. In this study, we demonstrated the validity, availability, and reliability of EBUS-TBNA for restaging both postsurgery and postchemoradiotherapy NSCLC. This method of evaluation displayed high diagnostic accuracy (93.2% and 100% for postsurgery and postchemoradiotherapy, respectively) and sensitivity (84.8% and 100% for postsurgery and postchemoradiotherapy, respectively). Based on our findings, EBUS-TBNA should be considered an effective and minimally invasive diagnostic procedure for restaging.

Until recently, MS was considered the benchmark technique for evaluating mediastinum. However, research and metaanalyses have shown that the sensitivity, specificity, NPV, PPV, and diagnostic accuracy of EBUS-TBNA are not only equivalent to those of the MS procedure in mediastinal staging but also displays a lower rate of complications. Therefore, in recent guidelines, EBUS-TBNA is recommended as the first choice for staging lung cancer [11]. Both MS and EBUS-TBNA have also been used for mediastinal reevaluation in NSCLC [7, 9, 20, 24–26]. However, both techniques have low sensitivity and poor diagnostic accuracy in restaging. Mediastinal scarring, which results from neoadjuvant therapy and prior surgery, is the most common handicap associated with remediastinoscopy [27]. De Waele et al. reported the sensitivity, specificity, and diagnostic accuracy of MS after neoadjuvant therapy in one of the largest studies of its kind (N = 104 patients); the results were 71%, 100%, and 84% for sensitivity, specificity, and diagnostic accuracy, respectively [28]. In other studies, similar MS performances were recorded: 61%–83% for sensitivity, 84%–91% for diagnostic accuracy, and 85% for NPV [24, 28–30]. However, MS is costly and requires general anesthesia and hospitalized treatment. It is also associated with several complications and high mortality rates. The morbidity of complications in MS is 0.6%–3%, the risk of hemorrhage is approximately 0.1%–0.6%, and mortality is approximately 0%–0.3% [31, 32]. Furthermore, hilar lymph nodes cannot be reached using MS.

The PET-CT technique is unreliable for assessing patients suspected of having cancer recurrence, as mentioned earlier. The rate of false positivity in PET-CT remains high, which is possibly related to postsurgical mediastinal changes and inflammation due to infection [18]. Therefore, pathological confirmation of PET-CT is mandatory. Yamamoto et al. reported 100% sensitivity, specificity, PPV, and NPV of EBUS-TBNA in the assessment of postoperative nodal recurrence in patients with lung cancer in a comparison study with PET-CT [18]. EBUS-TBNA has also been shown to have low sensitivity and diagnostic accuracy in mediastinal restaging, in the range of 50%–76% and 76%–89% [14, 21, 25,26]. However, Erdogan et al. reported the sensitivity and diagnostic accuracy of EBUS-TBNA in patients with Stage IIIA-N2 NSCLC at 82.1% and 88.6%, respectively [23]. In another study, Santos et al. found that the sensitivity and diagnostic accuracy of EBUS-TBNA in locoregional recurrence diagnosis were 80.9% and 86.6%, respectively [19]. Additionally, in a recent study, Bo Yan et al. found sensitivity and accuracy rates of the EBUS-TBNA diagnosis of recurrence in postsurgery patients with lung cancer to be 94.1% and 95%, respectively [20]. The results of our study were similar to those of these previous studies. In the postsurgery group, sensitivity and diagnostic accuracy were 84.8% and 93.2%, respectively, whereas all the values were satisfactory in the medically inoperable group. We thought that this difference was due to the presence of nonsampled and insufficiently sampled lymph nodes in the postsurgery group.

No serious complications were identified in any previous study. However, the values for restaging with EBUS-TBNA were lower than those of the initial mediastinal staging for NSCLC. Several factors may explain these differences. First, after chemotherapy and/or radiotherapy, lymph nodes that originally contained the tumor often began to undergo necrosis and fibrosis. These fibrotic lymph nodes may be more difficult to analyze through biopsy and may yield less cellular material for histological analysis. This phenomenon may explain the reason that in many EBUS-TBNA, lymph node tissue is sampled successfully, but no malignant cells are detected. Second, malignant cells may be focal within the node and/or may be located within areas of the dense extracellular matrix. Lastly, the presence of necrosis in the aspirated sample often hinders pathological interpretation [23]. Nonetheless, EBUSTBNA is an accurate, minimally invasive, and repeatable technique with lower complication rates than Med for restaging NSCLC. In addition, hilar lymph nodes can be sampled easily using EBUS-TBNA.

To date, only a limited number of studies available consisting of a small number of patients (generally 40–50 patients) have been published demonstrating the reliability of EBUS in restaging of postsurgical patients with high diagnostic accuracy. In addition, in published studies, follow-up periods are generally limited to 6 months. Unlike these studies in the literature, a large number of patients (n = 100), and lengthy follow-up (minimum 12, mean 20 months for all) were among our study’s prominent strongest ways. The present study has several limitations. Because this study was retrospective, we were unable to confirm EBUS-TBNA-negative patients using invasive procedures.

## 5. Conclusion

At present, knowledge about patients who are the most at risk for recurrence after curative treatment for lung cancer remains unclear. Moreover, there is a paucity of evidence regarding the role of surgical and endoscopic modalities in patients with advanced lung cancer. However, the results of our study confirmed that, because of its high sensitivity, NPV, and diagnostic accuracy, EBUS-TBNA should be considered a feasible, safe, and accurate procedure for reevaluation in both postsurgical and previously treated NSCLC patients.

## Figures and Tables

**Figure:**
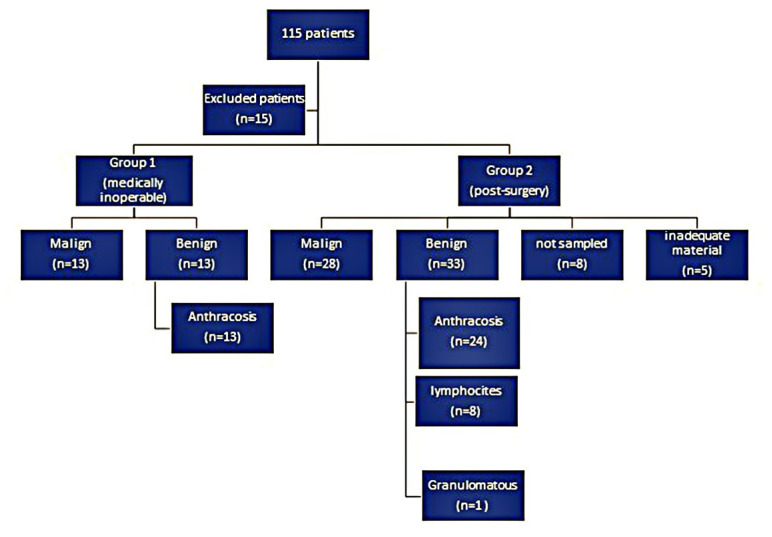
Flow chart of the study.

**Table 1:** Characteristics of patients.

Characteristics	Group 1 n (%)	Group 2 n (%)
Sex		
Male	21 (80.8)	69 (93.2)
Female	5 (19.2)	5(6.8)
Mean age (years)	63.9 ± 9.9	62 ± 7.9
Histology		
Squamous	18 (69.2)	34 (45.9)
Adenocarcinoma	6 (23.1)	38 (51.4)
NSCLC (not subtyped)	2 (7.7)	–
Adenosquamous	–	2 (2.7)
Chemotherapy	4 (15.4)	–
Chemoradiotherapy	22 (84.6)	–
Stage		
IA	–	11 (14.9)
IB	–	21 (28.4)
IIA	–	23 (31.1)
IIB	1 (3.8)	17 (23)
IIIA	19 (73.1)	2 (2.7)
IIIB	6 (23.1)	–
Surgery procedures	–	
Lobectomy	–	59 (79.7)
Pneumonectomy	–	12 (16.2)
Wedge resection	–	3 (4.1)
Median follow up time (months) (min–max)	14 (12–36)	18 (12–60)
Total	26 (100)	74 (100)

**Table 2:** Characteristics of punctured lymph nodes.

Group 1	Number of sampled lymph nodes	n (%)	Stations	n (%)	Node s ze (mean ± SD mm)

			4R	12 (46.2%)	10.5 ± 4.3
			4L	2 (7.7%)	12.5 ± 3.5
	1	18 (69.2%)	10R	1 (3.8%)	8
	2	7 (26.9%)	11R	3 (11.5%)	8.4 ± 4
	3	1 (3.8%)	11L	3 (11.5%)	9 ± 4.4
			7	12 (46.2%)	14.4 ± 6.1
			2L	2 (7.7%)	19.2 ± 1

Group 2			4R	26 (35.1%)	11.5 ± 6.1
			4L	16 (21.6%)	11.4 ± 6.7
	0	8 (10.8%)	10R	4 (5.4%)	11.5 ± 1.9
	1	34 (45.9%)	10L	1 (1.4%)	30
	2	27 (36.5%)	11R	11 (14.9%)	13.3 ± 6.5
	3	5 (6.8%)	11L	16 (21.6%)	9.9 ± 3.5
			7	24 (32.4%)	13.4 ± 8.5
			3P	1 (1.4%)	12
			2L	4 (5.4%)	11 ± 3.4

**Table 3:** Diagnostic performance of EBUS-TBNA.

	Group 1	Group 2
Sensitivitiy	100%	84.8%
Specificity	100%	100%
PPV	100%	100%
NPV	100%	89.1%
Diagnostic accuracy	100%	93.2%

PPV: Positive predictive value, NPV: Negative predictive value.
